# From threat to cure: understanding of virus-induced cell death leads to highly immunogenic oncolytic influenza viruses

**DOI:** 10.1038/s41420-020-0284-1

**Published:** 2020-06-11

**Authors:** Julijan Kabiljo, Johannes Laengle, Michael Bergmann

**Affiliations:** 1 Division of General Surgery, Department of Surgery, Comprehensive Cancer Center Vienna, Medical University of Vienna, Waehringer Guertel 18-20, 1090 Vienna, Austria; 2 Ludwig Boltzmann Institute Applied Diagnostics, Medical University of Vienna, Waehringer Guertel 18-20, 1090 Vienna, Austria

**Keywords:** Tumour immunology, Tumour virus infections, Cancer immunotherapy

## Abstract

Oncolytic viruses constitute an emerging strategy in immunomodulatory cancer treatment. The first oncolytic virus, Talimogene laherparepvec (T-VEC), based on herpes simplex virus 1 (HSV-1), was approved by the Food and Drug Administration (FDA) and European Medicines Agency (EMA) in 2015. The field of oncolytic virotherapy is still in its beginnings, since many promising viruses remain only superficially explored. Influenza A virus causes a highly immunogenic acute infection but never leads to a chronic disease. While oncolytic influenza A viruses are in preclinical development, they have not made the transition into clinical practice yet. Recent insights into different types of cell death caused by influenza A virus infection illuminate novel possibilities of enhancing its therapeutic effect. Genetic engineering and experience in influenza A virus vaccine development allow safe application of the virus in patients. In this review we give a summary of efforts undertaken to develop oncolytic influenza A viruses. We discuss strategies for targeting viral replication to cancerous lesions and arming them with immunogenic transgenes. We furthermore describe which modes of cell death are induced by influenza A virus infection and how these insights may be utilized to optimize influenza A virus-based oncolytic virus design.

## Facts

- Oncolytic influenza A virus displays selective replication in tumor cells.
- Influenza A virus can be targeted to tumors and armed with cytokines.
- Growth optimized influenza A virus for a phase I application can be generated.
- Oncolytic influenza A virus prototypes have been tested in humans as vaccine candidates and proven to be safe.

## Open questions

- Optimal viral subtypes as well, as the impact of preexisting anti-viral immunity may still be determined.
- Combination with other therapeutic modalities should be explored.
- Oncolytic effects on clinical outcome needs to be proven in prospective randomized clinical trials.

## Introduction

The observation of viral infections leading to the reduction of cancerous tumors was reported throughout the 20th century^1,2^. The first report of a complete tumor remission in the context of an influenza A infection dates back to 1904^3^. Preclinical models solidified the assertion of oncolytic effects of influenza A viruses^4,5^. However, the lack of understanding of virus biology made a safe and effective development of oncolytic A viruses impossible at that time. In the last decades advancements in molecular virology and viral engineering led to the clinical development of a variety of oncolytic viruses, including herpes simplex virus (HSV)^6^, reovirus^7^, vaccinia virus (VV)^8^, vesicular stomatitis virus (VSV)^9^, adenovirus^10^, newcastle disease virus (NDV)^11^, measles virus (MeV)^12^ and picornaviridae^13^. This renewed interest in oncolytic viruses resulted in the Food and Drug Administration (FDA) and European Medicines Agency (EMA) approval of the first oncolytic virus, Talimogene laherparepvec (T-VEC), in 2015^6^. T-VEC is an HSV, which is modified to grow selectively in tumor cells and express the immuno-stimulatory transgene granulocyte-macrophage colony-stimulating factor (GM-CSF). The development of oncolytic viruses was based on conditional replication of prototype viruses in malignant tissue, while being attenuated in normal tissue^14^. The lytic effect of the virus was initially thought to be the main principle of their anti-cancer activity. Later it turned out that the therapeutic effect was mainly promoted by a pro-inflammatory stimulation of the tumor immune microenvironment (TIME) counteracting tumor-associated immunosuppression. Thus, the concept of virotherapy largely overlapped with the rational principles of immune checkpoint inhibiting antibodies^15–22^. Importantly, the combination of oncolytic viruses with checkpoint inhibitors appears to be highly beneficial in a number of preclinical models^23–25^. In this line, a seminal paper by Zamarin et al. indicated, that an oncolytic NDV sensitized an immunologically “cold” murine tumor to systemic checkpoint inhibitors, which increased the rate of tumor remission^23^. Similarly, combining T-VEC with immune checkpoint inhibitors was associated with complete remission in 22% of stage IIIB/IV melanoma patients in a small phase I study strongly supporting this concept^26^. Those observations prompted a high level of interest in the field of virotherapy.

With respect to oncolytic influenza A viruses, only preclinical studies have been accomplished yet, in spite of the fact, that this virus family is well studied and known to be highly immunogenic. In this review we summarize major milestones in the development of oncolytic influenza A viruses. We delineate in which manner targeted replication in cancer cells was achieved. Furthermore, we discuss strategies to arm influenza A viruses with immuno-stimulatory transgenes. Finally, we discuss which types of cell death influenza A virus-infected cancer cells succumb to and their implications for future design of oncolytic A viruses.

There are several hallmarks viral candidates should possess in order to be considered for development into oncolytic agents: their genetics and biology should be well known and targeting to cancerous tissue needs to be feasible. They should be highly immunogenic and exert lytic activity leading to an immunogenic cell death (ICD) of malignant cells, while sparing normal tissue^27^. They should not lead to a chronic disease or retain capability of integrating into the human genome. Moreover, the use of the viruses as an oncolytic agent must be safe, in terms of excluding the possibility of the induction a pathogenic virus, which causes a disease the human population. It should also be feasible to genetically modify the virus and arm it with recombinant transgenes to enhance its immunogenicity or stimulate targeted anti-cancer mechanisms.

**Fig. 1:**
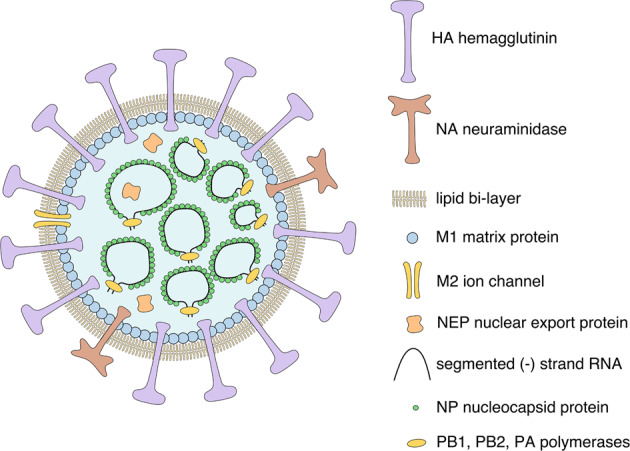
Components of the influenza A virus. Schematic representation of all components of the influenza A virus virion.

Utilizing an influenza A virus as an oncolytic agent has several advantages. The influenza virus is a small virus of the *Orthomyxoviridae* family, commonly known for causing the flu^28^. It comprises 4 genera, influenza A, B, C, and D viruses, type A being the most extensively studied one^28,29^. While the influenza virus can induce strong immunogenic reactions and intense pathology in humans, it never leads to chronic disease and attenuated forms have been described^30,31^. Influenza virus is an enveloped, negative-strand RNA virus with no reverse transcriptase or DNA integration activity^28^. These factors predispose it as an ideal vector for oncolytic therapy. Oncolytic virus development focused on influenza A virus. This virus subtype contains 8 separate RNA fragments, kept in cyclical conformation within the 80–120 nm large virion^28^. These segments encode 11 viral proteins necessary for viral structure and replication (Fig. 1), as well as the nonstructural protein 1 (NS1)^28^, which antagonizes the anti-viral reaction of the host^32^. The extensive knowledge and infrastructure that has previously been established for the production of seasonal influenza vaccinations reduces the amount of novel biotechnological engineering and regulatory issues, which are necessary for clinical development of the virus in the field of oncology^33^.

### The interplay of influenza virus and cell death

The rational development of a conditionally replicating phenotype of a virus in tumor tissue requires the understanding of virus-host interactions, particularly how viruses lyse infected cells and how cells protect themselves from the lytic infection. Influenza A viruses have been shown to induce multiple distinct modes of cell death^34^.

In the early phase of infection, the virally encoded protein NS1 inhibits apoptosis^35,36^, suggesting that apoptosis plays a role in anti-viral defense^37,38^. In the absence of NS1 apoptosis appears to be induced through the viral-RNA-mediated induction of retinoic acid-inducible gene I (RIG-I) and interferon (IFN) signaling including protein kinase R (PKR) and eukaryotic initiation factor 2 alpha (eIF2α) activation and subsequent block of translation^39–41^. NS1 has also been shown to inhibit apoptosis though interaction with the pro-apoptotic scribbled planar cell polarity protein (scribble)^42^.

However, influenza A viruses have a two-sided relationship to apoptosis^37^. There is evidence, that growth of influenza viruses is dependent on apoptosis^43^. Specifically, caspase 3 appears important for viral replication^44^. In this line, the influenza A virus can actively induce apoptosis. Apoptotic signaling may be initiated intrinsically through the viral protein PB1-F2^45^. A further major inducer of apoptosis during influenza A virus infections is the viral nucleoprotein (NP), interacting with the host’s Bcl-2-associated X protein (Bax) inhibitor clusterin, leading to Bax induced apoptosis^46^. Extrinsic induction of cell death, which inhibits viral replication at a late stage of viral life cycles, has been reported to occur through the release of tumor necrosis factor (TNF) receptor ligands, depending on nuclear factor kappa-light-chain-enhancer of activated B-cells (NF-κB) activation^47^. This process is counterbalanced by NF-κB inactivation through NS1^48^. The viral surface glycoprotein neuraminidase (NA) can also be involved in induction of cell death, as it enhances apoptosis through activation of transforming growth factor beta (TGF-β)^49^.

**Fig. 2:**
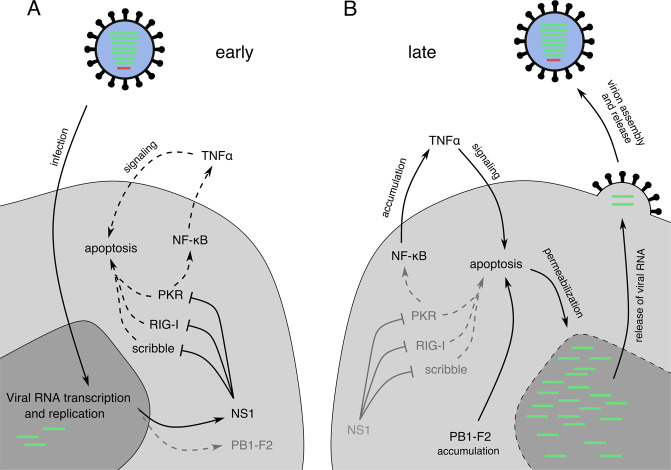
A proposed model of how influenza A virus controls apoptosis in 2 phases. **a** In the early phase of an influenza virus infection, the virus benefits from reduced apoptosis, in order to transcribe viral structural proteins and replicate viral RNA. Anti-apoptotic functions are mediated by NS1, interfering with various danger signaling proteins and reducing NF-κB induced TNFα release. **b** In the late phase of influenza virus infection, the pro apoptotic viral protein PB1-F2 and the weakened TNFα release might accumulate, leading to enhanced apoptotic signaling. Apoptosis enhances the release of viral RNA from the nucleus, a prerequisite for virus assembly and release. NF-κB, nuclear factor kappa-light-chain-enhancer of activated B cells; TNFα, tumor necrosis factor alpha.

There are multiple theories, why influenza A virus may actively induce apoptosis. Overall, there seems to be a fine, time-dependent balance of pro- and anti-apoptotic stimuli, which are tightly controlled by the virus. Upon overexpression of anti-apoptotic molecules influenza A virus titers are reduced due to viral RNA-protein complexes being retained in the nucleus^43,50^. Interestingly, caspase activation has been shown to enable diffusion of nuclear proteins into the cytoplasm^51^. This suggests that inhibition of both apoptosis and innate anti-viral responses through NS1 is necessary for viral propagation, especially in the initial phases of infection. In the late phase, activated caspases are needed to release viral RNA from the nucleus (Fig. 2). This theory is further reinforced by the observation, that the anti-mycotic amphotericin B enhances influenza virus growth^52^. Amphotericin B stabilizes pores within cellular membranes. This mechanism has been shown to aid RNA particles in passing through different cellular compartments^53^ and might also enable viral RNA release from the nucleus.

Influenza viral matrix-protein 2 (M2) and NS1 have been shown to induce autophagy^54^. This process leads to active transport of the danger associated molecular pattern (DAMP) adenosine triphosphate (ATP) out of the cell, resulting in immunogenic reactions^55,56^.

In conclusion, influenza A virus induces an apoptotic cell death through a number of pathways. It should be taken into account, that virally encoded danger molecules convert the apoptotic cell death into an ICD, leading to the stimulation of cytokines.

### Influenza A viruses can be targeted to malignant cells

In order to create a virus that initiates potent anti-cancer-immune responses without causing an infectious disease, it is necessary to target them to cancer cells. Mammalian cells react to viral infections by secreting type I IFN. Most viruses develop strategies to circumvent these immunogenic effects. It was shown, that the influenza A virus lacking NS1 (delNS1) generated by Egorov et al.^32^ was unable to replicate in mice with a functioning IFN signaling pathway^57^. However, it retained lethality in signal transducer and activator of transcription 1 (STAT1) knockout mice, which are unable to react to IFN^57^. Since many cancers downregulate components of the IFN signaling pathway^58–60^, attenuation of the NS1 protein appeared to be an attractive strategy for targeting growth of influenza A viruses to malignant tissue. We were able to show that deletions of various lengths within the NS1 gene of an influenza A/Puerto Rico/8/34 (PR8) based H1N1 virus yielded potent anti-cancer effects in IFN resistant human melanoma (SK-MEL1) xenografts^61^.

Another mechanism through which NS1 counteracts cellular responses to influenza A virus infection is the inhibition of the double-stranded RNA sensor PKR^36,62,63^. We demonstrated that NS1 deleted influenza A virus was lethal to PKR knockout mice but was not lethal in wild-type mice^36^. Oncogenic rat sarcoma (RAS) gene mutation, present in approximately a third of all cancer subtypes, results in PKR inhibition^64^. We showed that NS1 deletion targets a PR8 influenza virus towards RAS mutated tumors in a PKR dependent manner, using mouse xenografts of human 518 melanoma cells, transfected with oncogenic RAS^65^.

There are also developments of oncolytic influenza A viruses, which are based on attenuation markers other than the NS1 deletion. One preclinical study in murine non-small cell lung cancer (NSCLC) xenografts showed efficacy and safety of wild-type NS1, laboratory adapted, PR8 H1N1 virus^66^. Another study screened a variety of wild-type influenza A viruses for their infectivity in pancreatic carcinoma cell lines and showed oncolytic effectiveness in a mouse model of human pancreatic cancer^67^. Similarly, the seasonal flu vaccination has recently been assessed for sensitizing murine B16 melanoma models to immune check point inhibitor therapy, which showed promising results^24^.

### NS1-deleted (delNS1) viruses are potent stimulators of the immune system

**Fig. 3:**
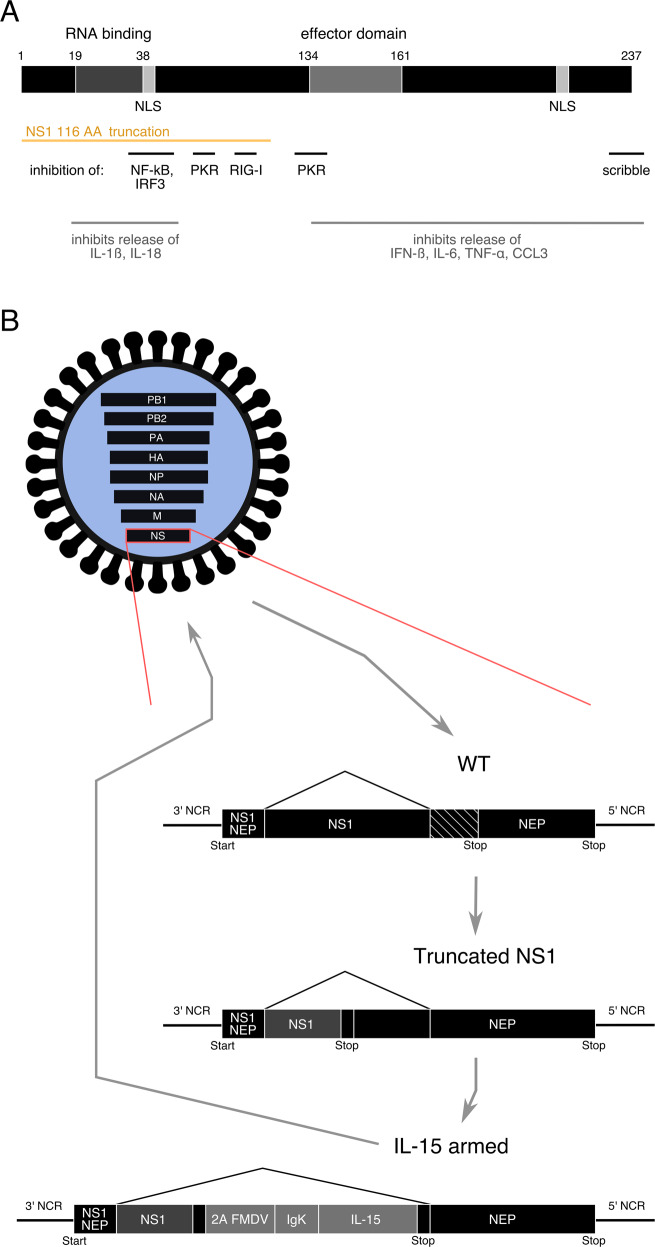
Functions of the influenza A virus NS1 protein and generation of an armed oncolytic influenza A virus. **a** Schematic representation of the NS1 protein. Major domains are represented and their AA positions indicated above. The yellow line represents parts of NS1 expressed after truncation to 116 AA. Inhibitory functions of NS1 relevant to oncolytic virus development and the domains they have been attributed to are indicated^42,48,62,63,68,72,75^. **b** Example of genetic modifications in an influenza virus designed to be used as oncolytic agent^94^. Modifications are carried out on the NS segment. In the first step NS1 is truncated to a length of 116 AA, leaving NEP intact. In a second step the armed transgene, in this example IL-15, is encoded in the reading frame of NS1. It is connected to an IgK and separated from NS1 with the H2 FMDV. Reading frames for NS1 and NEP have a common beginning. NS1 continues on, while an alternative reading frame is created for NEP through splicing. The short, frame shifted overlap of the end of NS1 and middle part of NEP is indicated by the diagonal hatching pattern. The complete NEP reading frame is generated through splicing in the attenuated and armed examples as well. AA amino acids, IL-15 Interleukin-15, NS1 nonstructural 1 protein, NEP nuclear export protein, IgK mouse-derived IgKappa signal peptide, 2A FMDV 2A cleavage site of the foot and mouth disease virus.

Apart from the conditionally replicating phenotype in malignant cells influenza A viruses with NS1 deletions are associated with a more potent stimulation of the innate immune system than wild-type viruses. This is due to the fact that the attenuation is linked to deletions in the viral inhibitor of the innate immune system. The inhibitory effect of NS1 on the immune system is based on its polyfunctional nature (Fig. 3a). With respect to cellular innate immune mediators, NS1 was shown to inhibit interferon regulatory factor 3 (IRF3) and NF-κB^48,68^. Since NS1 deleted influenza A viruses are capable of inducing strong PKR pathway upregulation, it seems plausible that known downstream signaling might lead to calreticulin (CALR) exposure on the cell membrane^69^. Here CALR acts as a DAMP, causing enhanced immunogenicity of apoptotic bodies after influenza A virus infection in PKR sensitive cancers^70^.

A transcriptional profiling confirmed that a delNS1 virus infection caused a much more potent induction of an immuno-stimulatory gene pattern compared with wild-type virus^71^. Correspondingly, infection of macrophages with the delNS1 virus is not only associated with an ICD, but also with a more potent stimulation of innate cytokines as compared with wild-type viruses^72^. The removal of C-terminal domains of NS1 provoked release of type I IFN, interleukin 6 (IL-6), tumor necrosis factor α (TNF-α), and chemokine (C–C motif) ligand 3 (CCL3)^72^. Removal of N-terminal domains induced release of interleukin 1β (IL-1β) and interleukin 18 (IL-18)^72^.

Importantly, whereas partial truncation of NS1 increased upregulation of pro-inflammatory genes, the full NS1 deletion leads to further increased expression^71^. The length of the NS1 deletion correlated with the potency of its immune stimulation and the level of its attenuation^31,32^. Full NS1 deletion results in viruses characterized by an abortive infection. Thus, the level of pro-inflammatory immune stimulation and the level of attenuation can be titrated by the length of the deletion. Correspondingly, we observed that partial NS1 deletion to 80 or 116 remaining amino acids (AA; removing the effector domain, but leaving the RNA binding domain and one nuclear locating sequence) induced an optimal balance between attenuation and the oncolytic effect^61,73,74^. This truncation should reduce PKR pathway inhibition while retaining inhibition of immunogenic signaling through the RIG-I pathway^62,63,75^ (Fig. 3a). Both PKR and RIG-I have been implicated in type I IFN production^76,77^. They both recognize the short double-stranded RNA “panhandle”, which holds the single stranded RNA segments of the influenza A virus genome in a circular conformation^78–81^.

The immunogenic character of the delNS1 virus had also been explored in the context of potential vaccination agents. In a preclinical study the use of complete or partial NS1 deleted H1N1, H3N2, and H5N1 subtypes as vaccines induced potent T cell and B cell responses, protecting mice and ferrets against influenza A virus challenge^31,82^. Importantly, the virus did not cause any disease in mice or the primate *Macaca mulatta* when applied intranasally, corresponding to systemic application^82^. In a phase I clinical trial we evaluated a NS1-deleted H1N1 virus as an intranasal vaccination vector^83^. Another clinical trial conducted a similar study using the H5N1 virus^84^. These vaccine studies are relevant for the development of NS1-deletion viruses as oncolytic agents since they indicated that NS1-deletion viruses are safe in humans and are able to stimulate a potent adaptive immune response at the same time.

Certainly, the major requirement of oncolytic viruses is the stimulation of an adaptive T cell response against tumor-associated antigens (TAA). In this line, we and others have demonstrated, that NS1-deletion viruses can be employed to stimulate dendritic cells (DC) to mount a T cell response against malignant cells via cross presentation^85,86^. Stimulation of T cells can either be induced by DCs exposed to delNS1 virus generated virolysates or by exposing DCs infected with delNS1 viruses to tumor cell lysates. Again, partial NS1-deletion viruses were more effective than full deletion of the NS1 protein. Those *ex vivo* assays might somewhat model the in vivo tumor microenvironment during oncolytic therapy. Exposure of immune cells to oncolytic NS1 deletion viruses was also shown to induce direct IFN dependent cytotoxic effects of peripheral blood mononuclear cells (PMBCs), including T cells, B cells, monocytes and natural killer (NK) cells against various cancer cell lines^87^. These cytotoxic effects might contribute to the viruses’ therapeutic effect in the tumor microenvironment.

### Targeting influenza viruses to tumor cells by viral entry

Influenza A virus entry depends on a protease to cleave the hemagglutinin (HA), a protein mediating viral entry. Thus, the presence of trypsin or equivalent proteases in the tissue is substantial for host restriction. It restricts the influenza A virus infection to the lung, the enteric system and for some viral isolates to the brain. We discovered that various colon cancer cell lines express trypsin and allow oncolytic influenza A virus growth in the absence of exogenous protease^73^. To further target the influenza A virus to tumor tissue we generated a virus in which the conformational change of HA, which enables viral entry, relies on the protease elastase^74^. Elastase has been shown to be present in tumor tissue due to expression by neutrophils. Elastase is also strongly expressed by malignant cells of pancreatic origin. Replacing the trypsin cleavage site with an elastase cleavage site has been shown to attenuate influenza A virus replication in swine and mice^88–90^. We therefore exchanged the trypsin cleavage site within the partially NS1 deleted (116AA) PR8 influenza virus to elastase. Elastase-dependent viruses yielded a potent therapeutic efficacy in murine melanoma (B16) and pancreatic ductal adenocarcinoma (PANC-1) xenograft models^74^. Thus, this attenuation marker present in HA can be used to target virus to tumor tissue and might allow for the use of HA which is currently not present in the human population, including the H7 or H9 subtypes.

### Arming oncolytic influenza viruses to enhance immunogenicity

Many clinical and preclinical studies have shown effectiveness of “non-armed” oncolytic viruses in cancer treatment. Still, their immuno-stimulatory properties do not always result in the expected potent anti-cancer effect. In order to optimize anti-cancer activity viruses are engineered to express various immuno-stimulatory transgenes, most prominently T cell and DC activating cytokines like interleukin-2 (IL-2), interleukin-15 (IL-15) or GM-CSF^91^. Within the influenza A virus background we were able to establish potent expression of various transgenes from a deleted or truncated NS1 reading frame, including the reporter gene green fluorescing protein (GFP), the cytokine IL-2, or CC-chemokine ligand 20 (CCL20)^92,93^. Using the viruses’ inherent property of a high mutational frequency we were able to adapt viruses to stably express transgenes up to 441 AA from the NS segment, together with nuclear export protein (NEP) and NS1 truncated to 116 AA^92^ using initial selection pressure. It seems likely that very long transgenes might be deleted, but the maximum transgene loading capacity has not yet been tested in influenza viruses.

We demonstrated superior therapeutic efficacy and enhancement of NK cell and T cell activation and proliferation when the partially NS1 deleted (116) influenza virus was armed with IL-15^94^ (Fig. 3b) in murine models. Subsequently, Penghui et al. were able to show anti-cancer activity of an NS1-deleted influenza A virus armed with GM-CSF in a human Hep-G2 liver cancer cell line xenograft model^95^. Hamilton et al. expressed a recombinant humanized cytotoxic T-lymphocyte-associated protein 4 (CTLA4) immune checkpoint inhibiting antibody from two different RNA fragments of the influenza A virus genome in order to enhance its anti-cancer effectiveness in a murine B16 melanoma model^96^.

In order to elicit specific immunological memory against known cancer epitopes, Efferson et al. established a combined strategy of oncolytic influenza A viruses armed with a vaccination peptide against the human epidermal growth factor receptor 2 (HER2)^97^. This approach led to potent initiation of effector and memory T cells in an in vitro DC-based assay.

Overall, multiple promising transgenes that enhance therapeutic outcomes in preclinical models have been postulated for oncolytic influenza A viruses.

### Safety aspects of oncolytic influenza viruses

**Fig. 4:**
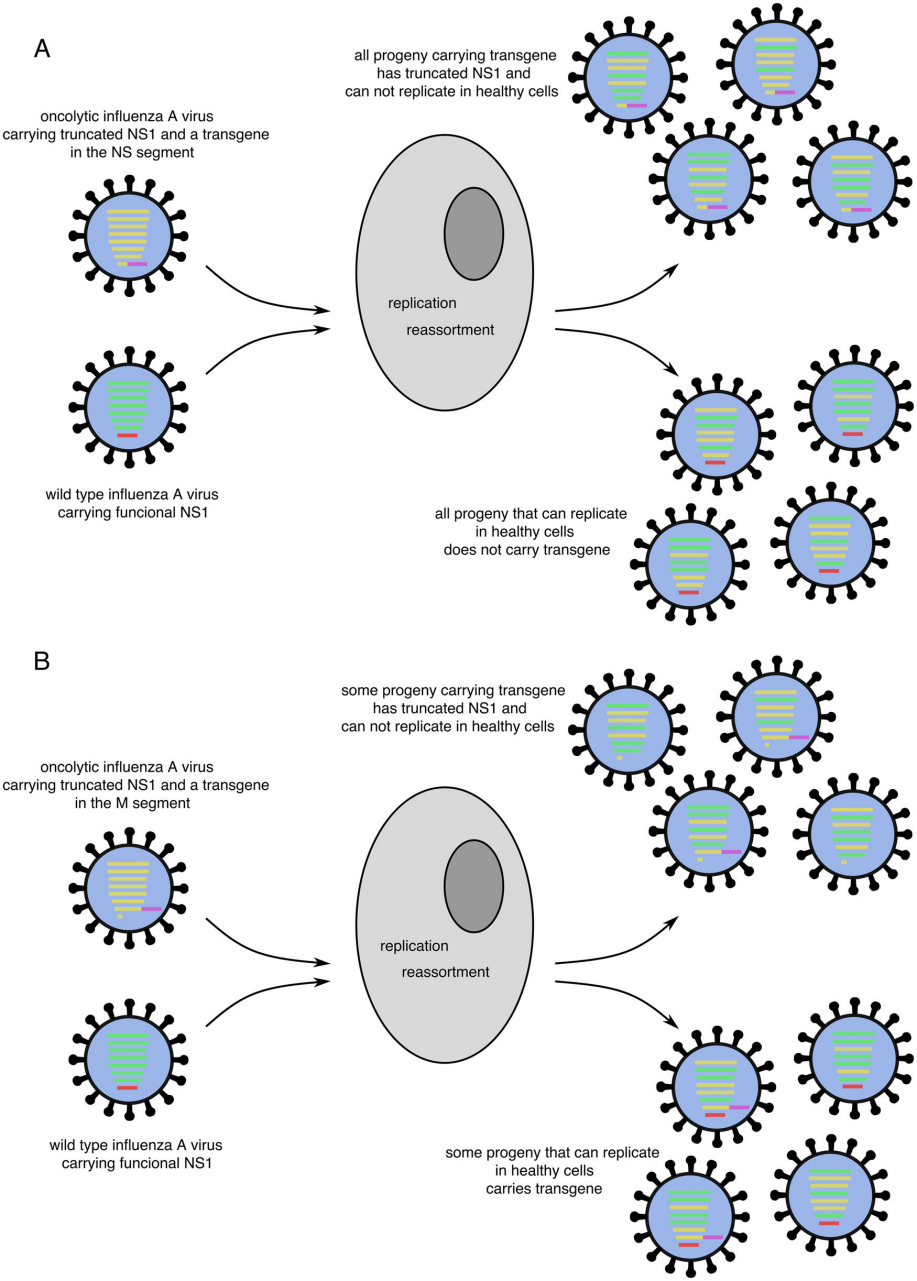
Reassortment of oncolytic influenza A viruses and wild-type influenza A viruses. Examples of reassortment of an armed oncolytic influenza virus attenuated by NS1 truncation during co-infection with wild-type influenza virus. **a** represents transgene expression from the NS1 reading frame. All reassortants carrying the transgene are attenuated. **b** represents transgene expression from the M segment. Some reassortants may carry both wild-type elements of the attenuation marker NS1 and a transgene, leading to unpredictable effects of the newly created virus. NS1 nonstructural 1 protein.

Influenza viruses remain a major health concern as they cause epidemics and pandemics. This happens due to the emergence novel reassortments as well, as novel mutants, generated by genetic changes caused by viral polymerases characterized by a low fidelity. Therefore, the use of the virus as a therapeutic agent requires specific safety attention. Any oncolytic virus candidate needs to be tested for its stable attenuation. The existence of licensed live influenza A virus vaccines clearly indicates, that the genetic stability of attenuated live influenza viruses is feasible. We could demonstrate that delNS1 viruses, including armed versions, can be safely passaged for more than 5 times, without losing their transgene or phenotype^83,92^. Moreover, expressing a transgene from a truncated NS1 reading frame within the NS segment, which codes for the attenuation marker at the same time, prevents the unwanted transmission of the transgene to a wild-type virus (Fig. 4). This is particularly desirable, since reassortment of the chimeric segment with wild-type segments of other influenza virus subtypes might lead to an unexpected pathogenic phenotype. Thus, in case transgenes are expressed within a wild-type segment, the apathogenic character of such chimeric viruses may need to be proven within the background of various subtypes.

### Enhancing oncolytic activity of the influenza A virus by optimizing immunogenic cell death – potential strategies

One strategy to optimize oncolytic virus-induced cell death is to enhance induction of apoptosis of neighboring cells. This can be achieved by arming the virus with a pro-apoptotic cytokine such as interleukin-24 (IL-24). We have shown that IL-24 sensitizes cancer cells to apoptosis within the background of an influenza A virus infection^98^. Importantly the apoptotic effect of IL-24 was strongly dependent on a second signal, namely the activation of the toll-like receptor 3 (TLR3) by viral RNA. This dependency targets the strong induction of apoptosis achieved by IL-24 to the tumor microenvironment, as the attenuated influenza virus used in this experiment cannot replicate otherwise. However, an influenza virus armed with IL-24 might be difficult to translate to clinical studies, due to IL-24 inhibiting viral growth and leading to low production titers^99^.

Another strategy to optimize oncolysis is the induction of a more immunogenic form of cell death such as necrosis or necroptosis^100^. Necroptosis is a form of programmed death a cell may engage in as an alternative to apoptosis depending on abundance of caspases and various signaling proteins within the cell. It is dependent on receptor-interacting serine/threonine-protein kinase 3 (RIPK3) activation and subsequent membrane lysis through mixed lineage kinase domain-like pseudokinase (MLKL) activation. This leads to a phenotype resembling necrosis. Necroptosis is considered to be highly immunogenic due to passive release of a large variety of DAMPs and cancer neo-antigen^101^. A recent report implicates necroptosis signaling through RIPK3 in inducing potent anti-tumor immune responses independent of the subsequent necrotic phenotype induced by MLKL and DAMP release^102^. Influenza A virus infection can be sensed by the cell through Z-DNA binding protein 1 (ZBP1). This can lead to either necroptosis or apoptosis^103^. Conversely, the cellular inhibitor of apoptosis protein 2 (cIAP2) protein has been shown to protect against influenza A virus-induced necroptosis^104^.

Necroptosis but also necrosis might be induced by specific influenza subtypes. Examples are the avian H5N1 and a reconstructed 1908 pandemic H1N1^105,106^. Similarly, H5N1 has been shown to inhibit apoptosis, which possibly enables the cell to undergo necroptosis^107^. H5N1 was also more potent than H1N1 in initiating cytokine responses^108^. Specifically, H5N1 seems to induce stronger chemotactic signals and thus stronger modulation of chemoattraction of immune cells^109^. Hartmann *et al*. recently described varying potential of different flu virus strains to cause necroptosis, specifically describing seasonal NC/99 H1N1 influenza A viruses as inducers and pandemic Cal/09 as inhibitors of necroptosis^110^. HA seemed particularly important in deciding cell fate in this experiment. These results indicate that choosing influenza A virus vectors according to their capability to initiate necroptosis is a promising strategy for future oncolytic virus design.

The influenza A virus NS1 protein can directly cause necroptosis through interaction with MLKL^111^. This form of necroptosis may be inferior to upstream pathway activation, since RIPK3 is necessary for strong immunogenic responses to necroptosis in cancer^102^. On the other hand, RIPK3 mediated effects seemed to depend on NF-κB activation in the same study. Infection with delNS1 virus may lead to similar effects, as it strongly induces NF-κB activation^48^.

Using in vitro models, we were not able to show necroptotic cell death after infection with delNS1 or wild-type viruses^98^. This may have been due to the lack of RIPK3 expression in the cancer cell lines examined. It is well established, that RIPK3 is downregulated in a variety of cancers^112^. This is usually correlated with worsened prognosis^112^. Interestingly, murine models indicate that necroptotic non-cancerous cells within the tumor microenvironment contribute to positive outcomes at least as much as necroptotic cancer cells^102^. Therefore, the potential of candidate oncolytic viruses to induce necroptosis in stroma cells should be examined.

A novel pathway of regulating immunogenicity in viral infections is oxeiptosis^113^. Viruses like influenza A virus generate radical oxidative species (ROS). Cells can sense ROS through Kelch-like ECH-associated protein 1 (KEAP1) which produces a dose-dependent effect^113^. At low doses of ROS the transcription factor nuclear factor erythroid 2-related factor 2 (NRF2) is activated by KEAP1 and promotes cell survival^113^. Also, KEAP1 binds to PGAM family member 5 (PGAM5), and consequently inactivates it. At higher doses, KEAP1 dissociates from PGAM5, leading to induction of oxeiptosis, a caspase-independent, immune-silent form of cell death^113^. Engaging in this type of cell death protects the cell from undergoing more immunogenic forms of death like necroptosis. PGAM5 knockout mice were shown to react to influenza A virus infection with enhanced necrotic histology and rapid death^113^. A malignant tumor might protect itself from ROS induced immunogenic forms of cell death through intact oxeiptotic signaling, which potentially reduces the effectiveness of oncolytic viruses. Downregulation of oxeiptotic cell death in the tumor microenvironment may be a promising strategy to enhance oncolytic virotherapy.

Further effects of oncolytic influenza A viruses on the cancer-immune microenvironment shown in murine models include activation of NK-cells and macrophage polarization towards immuno-stimulatory M1 phenotypes^66,114^. Recent evidence suggests NS1 deleted influenza virus to exhibit enhanced growth when combined with an IFN blocking agent in vitro^115^. Therefore, co-injection with such an agent may lead to intensified initial growth and enhanced subsequent reactions against the tumor. Oncolytic viruses have been combined with multiple agents in preclinical studies, like histone deacetylase inhibitors or the HER2 antibody trastuzumab^116,117^. Such novel combinations may prove to activate immunological anti-cancer mechanisms like antibody-dependent, cell-mediated cytotoxicity (ADCC), which is reduced in breast cancer patients^118,119^. The clinically approved oncolytic virus T-VEC is experimentally combined with other therapeutic modalities, such as radiotherapy^120^ or immune checkpoint blockade^26,121^. Similar methods may prove to be successful in influenza virus-based oncolytic virotherapy.

## Conclusion and outlook

Influenza viruses attenuated by means of truncated or deleted NS1 or by other attenuation markers have proven to be safe vaccines in clinical studies and have promising characteristics for use as oncolytic agents. Various factors governing their immunogenicity have been described, and strategies for optimizing their oncolytic effects established. Arming viruses with cytokines or checkpoint inhibitors appears to enhance their therapeutic effect. These insights into the immunomodulatory properties of such viruses may help further refine choices and design of viruses to be used in clinical trials. The future challenge is bringing this promising novel immunotherapeutic agent into clinical routine. Open questions remain the impact of preexisting immunity and combination with other anti-cancer drugs.
